# The impact of educational status on the clinical features of major depressive disorder among Chinese women

**DOI:** 10.1016/j.jad.2011.06.046

**Published:** 2012-02

**Authors:** Zhaoyu Gan, Yihan Li, Dong Xie, Chunhong Shao, Fuzhong Yang, Yuan Shen, Ning Zhang, Guanghua Zhang, Tian Tian, Aihua Yin, Ce Chen, Jun Liu, Chunling Tang, Zhuoqiu Zhang, Jia Liu, Wenhua Sang, Xumei Wang, Tiebang Liu, Qinling Wei, Yong Xu, Ling Sun, Sisi Wang, Chang Li, Chunmei Hu, Yanping Cui, Ying Liu, Ying Li, Xiaochuan Zhao, Lan Zhang, Lixin Sun, Yunchun Chen, Yueying Zhang, Yuping Ning, Shenxun Shi, Yiping Chen, Kenneth S. Kendler, Jonathan Flint, Jinbei Zhang

**Affiliations:** a No. 3 Affiliated Hospital of Sun Yat-sen University, No. 600 Tian He Road, Tian He District, Guangzhou, Guangdong 510630, PR China; b Wellcome Trust Centre for Human Genetics, Oxford OX3 7BN, UK; c Fudan University affiliated Huashan Hospital, No. 12 Wulumuqi Zhong Road, Shanghai 200040, PR China; d Shanghai Jiao Tong University School of Medicine affiliated Shanghai Mental Health Centre, No. 600 Wan Ping Nan Road, Shanghai 200030, PR China; e Shanghai Tongji University affiliated Tongji Hospital, No. 389 Xinchun Road, Shanghai 200065, PR China; f Nanjing Brain Hospital, No. 264 Guangzhou Road, Nanjing, Jiangsu 210029, PR China; g No. 4 Affiliated Hospital of Jiangsu University, No. 246 Nan Men Da Street, Zhenjiang, Jiangsu 212001, PR China; h Tianjin Anding Hospital, No. 13 Liu Lin Road, Hexi District, Tianjin 300222, PR China; i Shandong Mental Health Center, No. 49 East Wenhua Road, Jinan, Shandong 250014, PR China; j No. 1 Hospital of Medical College of Xian Jiaotong University, No. 277 West Yan Ta Road, Xi'an, Shaanxi 710061, PR China; k No. 1 Hospital of Zhengzhou University, No. 1 East Jianshe Road, Zhengzhou, Henan 450052, PR China; l No. 1 Mental Health Center Affiliated Harbin Medical University, No. 23 You Zheng Jie, Nangang District, Harbin, Heilongjiang, PR China; m Mental Health Center of West China Hospital of Sichuan University, No. 28 Dian Xin Nan Jie, Wu Hou District, Chengdu, Sichuan 610041, PR China; n Beijing Anding Hospital, Capital Medical University, No. 5 An Kang Hutong Deshengmen wai, Xicheng District, Beijing 100088, PR China; o Hebei Mental Health Center, No. 572 Dongfeng Road, Baoding, Hebei 071000, PR China; p Shengjing Hospital of China Medical University, No. 36 Sanhao Street, Heping District Shenyang, Liaoning 110817, PR China; q Shenzhen Kangning Hospital, No. 1080, Cui Zu Street, Luo Hu, Shenzhen 518020, PR China; r No. 1 Hospital of Shanxi Medical University, No. 85 Jiefang South Road, Taiyuan, Shanxi 030001, PR China; s Mental Hospital of Jiangxi Province, No. 43 Shangfang Road, Nanchang, Jiangxi 330029, PR China; t The First Affiliated Hospital of Jinan University, No. 613 West Huangpu Avenue, Guangzhou 510630, PR China; u Wuhan Mental Health Center, No. 70, You Yi Road, Wuhan 430022, PR China; v No. 3 Hospital of Heilongjiang Province, No. 135 Jiao Tong Lu, Beian, Heilongjiang, PR China; w Jilin Brain Hospital, No. 98 Zhong Yang Xi Lu, Siping, Jilin 136000, PR China; x The First Hospital of China Medical University, No. 155 Nanjing Bei Jie, He Ping District, Shenyang 110001, PR China; y Dalian No. 7 People's Hospital & Dalian Mental Health Center, No. 179 Ling Shui Lu, Gan Jing Zi District, Dalian, PR China; z The First Hospital of Hebei Medical University, No. 89 Donggang Road, Shijiazhuang 050031, PR China; aa Lanzhou University Second Hospital, Second Clinical Medical College of Lanzhou University, No. 82, Cui Ying Men, Lanzhou, Gansu 730030, PR China; ab Psychiatric Hospital of Henan Province, No. 388 Jian She Zhong Lu, Xinxiang, Henan, PR China; ac The Fourth Military Medical University affiliated Xijing Hospital, No. 17, Changle West Road, Xi'an, Shaanxi 710032, PR China; ad No. 4 People's Hospital of Liaocheng, No. 47 Hua Yuan Bei Road, Liaocheng, Shandong 252000, PR China; ae Guangzhou Brain Hospital/Guangzhou Psychiatric Hospital, No. 36 Ming Xin Lu, Fang Cun Da Dao, Li Wan District, Guangzhou 510370, PR China; af Clinical Trial Service Unit, Richard Doll Building, Old Road Campus, Roosevelt Drive, Oxford OX3 7LF, UK; ag Virginia Commonwealth University, Department of Psychiatry, Virginia Institute for Psychiatric and Behavioral Genetics, Richmond, VA 23298-0126, USA

**Keywords:** Major depressive disorder, Education, Socio-economic status, Symptom

## Abstract

**Background:**

Years of education are inversely related to the prevalence of major depressive disorder (MDD), but the relationship between the clinical features of MDD and educational status is poorly understood. We investigated this in 1970 Chinese women with recurrent MDD identified in a clinical setting.

**Methods:**

Clinical and demographic features were obtained from 1970 Han Chinese women with DSM-IV major depression between 30 and 60 years of age across China. Analysis of linear, logistic and multiple logistic regression models were used to determine the association between educational level and clinical features of MDD.

**Results:**

Subjects with more years of education are more likely to have MDD, with an odds ratio of 1.14 for those with more than ten years. Low educational status is not associated with an increase in the number of episodes, nor with increased rates of co-morbidity with anxiety disorders. Education impacts differentially on the symptoms of depression: lower educational attainment is associated with more biological symptoms and increased suicidal ideation and plans to commit suicide.

**Limitations:**

Findings may not generalize to males or to other patient populations. Since the threshold for treatment seeking differs as a function of education there may an ascertainment bias in the sample.

**Conclusions:**

The relationship between symptoms of MDD and educational status in Chinese women is unexpectedly complex. Our findings are inconsistent with the simple hypothesis from European and US reports that low levels of educational attainment increase the risk and severity of MDD.

## 1 Introduction

Epidemiological studies of major depressive disorder (MDD) support an inverse association between the prevalence of MDD and level of education (Lorant et al., 2003). Furthermore, previous studies have shown the potential impact of gender (Ross and Mirowsky, 2006), race (Kessler et al., 2005) and age (Mirowsky and Ross, 1992) on the association between educational status and clinical features of depression. Much less frequently investigated however has been the association between educational status and clinical features of depression as seen in clinical settings.

In this study we explore the relationship between educational status and a rich set of clinical features of MDD assessed in 1970 clinically ascertained cases of recurrent MDD in Han Chinese women. We sought to answer the following questions: first, does educational status predict MDD? Second, are there differences in the severity of illness among MDD patients with differing levels of education? Third, does the symptomatology or course of illness of MDD correlate with variation in educational level? Finally, are there differences in comorbidity among MDD patients with varying levels of education?

## 2 Methods

### 2.1 Samples

Data for the present study draw upon the ongoing China, Oxford and VCU Experimental Research on Genetic Epidemiology (CONVERGE) study of MDD. Analyses were based on a total of 1970 cases recruited from 53 provincial mental health centers and psychiatric departments of general medical hospitals in 41 cities in 19 provinces. All cases were female and had four Han Chinese grandparents. Cases were excluded if they had a pre-existing history of bipolar disorder, any type of psychosis or mental retardation. Cases were aged between 30 and 60, had two or more episodes of MDD, with the first episode occurring between 14 and 50 and had not abused drug or alcohol before the first episode of MDD. The mean age (and SD) of cases was 45.1 (8.8). All subjects were interviewed using a computerized assessment system, which lasted on average two hours for a case. All interviewers were trained by the CONVERGE team for a minimum of one week in the use of the interview. The interview includes assessment of psychopathology, demographic and personal characteristics, and psychosocial functioning. The study protocol was approved centrally by the Ethical Review Board of Oxford University and the ethics committees in participating hospitals in China.

### 2.2 Measures

The diagnoses of depressive (dysthymia and major depressive disorder) and anxiety disorders (generalized anxiety disorder, panic disorder with or without agoraphobia) were established with the Composite International Diagnostic Interview (CIDI) (WHO lifetime version 2.1; Chinese version), which classifies diagnoses according to the Diagnostic and Statistical Manual of Mental Disorders (DSM-IV) criteria (American Psychiatric Association, 1987). The interview was originally translated into Mandarin by a team of psychiatrists in Shanghai Mental Health Centre, with the translation reviewed and modified by members of the CONVERGE team. Phobias, divided into five subtypes (animal, situational, social and blood-injury, and agoraphobia), were diagnosed using an adaptation of the DSM-III criteria requiring one or more unreasonable fears, including fears of different animals, social phobia and agoraphobia, that objectively interfered with the respondent's life. The section on the assessment of phobias was translated by the CONVERGE team from the interview used in the Virginia Adult Twin Study of Psychiatric and Substance Use Disorders (VATSPUD) (Kendler and Prescott, 2006).

Education was treated in two ways: as the number of years of completed full time education and as an ordinal variable divided into seven levels: no education or pre-school (scored 0) primary school or below (scored 1), junior middle school (scored 2), senior middle school or Technical and vocational school (in China, they both take the same time to be finished) (scored 3), Adult/radio/television schooling, evening education or junior college (scored 4), bachelor degree (scored 5), and master degree or above (scored 6).

All interviews were carried out using a computerized system developed in house in Oxford and called SysQ. Skip patterns were built into SysQ. Interviews were administered by trained interviewers and entered offline in real time onto SysQ, which is installed in the laptops. Once an interview is completed, a backup file containing all the previously entered interview data can be generated with database compatible format. The backup file together with an audio recording of the entire interview is uploaded to a designated server currently maintained in Beijing by a service provider. All the uploaded files in the Beijing server are then transferred to an Oxford server quarterly.

### 2.3 Statistical analysis

Statistical analyses were performed using the software package SPSS 17.0 (SPSS Inc., Chicago, IL). We performed linear and logistic regression analyses to estimate the association between years of education with MDD and comorbid disorders. Coefficient values, odds ratios and 95% confidence intervals were used to quantify the strength of associations. The statistical significance for all tests was set at P < 0.05 and corrected where necessary for multiple testing using a Bonferroni correction.

## 3 Results

We compared the educational status of cases and controls and found that 52% of cases had completed no more than middle school education, compared to 47% of controls. We estimated the effect of education on the risk of MDD by logistic regression, after scoring each subject for the number of years of education obtained. There was no significant effect of years of education on the risk of developing MDD. However the odds ratio was slightly above 1 (1.013), and since this is a value for each year of education, a larger effect might be observed in those who have spent more time in school. We found that those with more than 10 years of education do indeed have a higher odds ratio of 1.14 (95% confidence intervals: 1.07–1.22, P < 0.00001).

We looked next at the effect of education on the symptomatology of MDD in cases only. We first scored each MDD patient for the presence of each of nine diagnostic MDD “A” criteria and regressed this total on their educational status (ranked from 1 to 7). In this, and subsequent analyses age of the patient was incorporated as a covariate. We found that increasing criteria count scores were significantly associated with decreasing years of education (P < 0.001) with an adjusted R^2^ of 0.24. We examined the relationship again using a broader list of 26 depressive symptoms. Again we found an inverse relationship between educational status and total number of symptoms endorsed (P < 0.001, R^2^ = 0.106).

We sought to determine if years of education was related to specific depressive symptoms. Results from multivariate binary logistic regression between each symptom and education status are shown in Table 1. Applying a Bonferroni corrected significance threshold of 0.0019 (0.05/26), five symptoms were significantly associated. Of note, at an experiment-wise level, the association between individual symptoms and years of education was highly significant, with 18 out of 26 symptoms associated with a P < 0.05, where only slightly more than one would be expected by chance.

Patients with fewer years of education are significantly more likely to experience neurovegetative (loss of sleep and appetite), emotional (irritability, unhappiness, crying and anxiety), cognitive (guilt, worthlessness), and psychomotor symptoms (slow speech or movement) as well as suicidal ideation. By contrast patients with higher educational status are more likely to experience hypersomnia and apathy.

We looked at the association between educational status and the course of MDD. We found that the age of onset of MDD is likely to be later for those cases with more years of education (F-statistic: 84.1 (1,1913), P < 0.001), but the effect is modest (the estimate is − 0.08). We found no significant association between educational status and either the number of episodes (P = 0.1) or the duration of the longest episode of MDD (P = 0.2).

Finally we examined the association of educational status with risk for six anxiety disorders, dysthymia and melancholia (Table 2). After applying a Bonferroni correction for multiple testing (P < 0.005), we found one significant result: subjects with lower levels of education were at increased for melancholia.

## 4 Discussion

We investigated the relationship between educational status and features of major depression in almost 2000 Han Chinese women, diagnosed with recurrent MDD and identified in 53 hospitals across China. Based on the published Western literature, we hypothesized that among women with recurrent MDD, lower educational attainment would be associated with a more severe depressive syndrome, with greater numbers of symptoms, more episodes, higher co-morbidity and an earlier age of onset (Alonso et al., 2004; Bijl et al., 1998; Kessler et al., 2005; Lorant et al., 2003; Mirowsky and Ross, 1992; Ross and Mirowsky, 2006; Weiss et al., 2009).

Our findings only partly support this view. Rather than being protective, more years of education is a risk factor for recurrent MDD in Chinese women. We did not find low educational status to be associated with an increase in the number of episodes, or increased rates of co-morbidity with anxiety disorders. Furthermore the association between age of onset and education attainment was opposite to that predicted, with an earlier age of onset associated with higher educational attainment. Our results show that educational status is important in modifying some aspects of the depressive picture in women in China. However, the relationship is not a simple one. In Birnbaum's terminology, educational status appears to be a pathoplastic influence on major depression (Birnbaum, 1974).

Most epidemiological studies report a significant, but relatively small effect of the number of years of education on the risk of MDD. While higher education was associated with lower rates of mood disorder in a pan-European study (Alonso et al., 2004), and in Holland those with the fewest years of education had the highest morbidity rates (Bijl et al., 1998), in Finland no significant difference between different educational groups was found (however the OR between the lowest and highest income groups, which is correlated with educational attainment, was 1.93) (Lindeman et al., 2000). In the National Comorbidity Survey the association of educational status was largely confined to predicting highly comorbid MDD (Kessler et al., 2005). By contrast, a prior study in a South Western city in China reported a very strong association (odds ratio of 18.84) between MDD and those with the fewest year of schooling (Lu et al., 2008). One explanation for these findings is that the effect of education may depend on the group it affects. For example Coryell et al. (1992) report that while men with no college education were more likely to develop MDD than men with college education, the opposite was true for women. Differences in ethnicity may also contribute to the consequences educational status on susceptibility to MDD (Gavin et al., 2010; Kessler et al., 2003).

We find that the effect of education impacts differentially on the symptoms of depression. To our knowledge this has not been examined before. Lower educational attainment is associated with more neurovegetative symptoms and increased suicidal ideation and plans. MDD patients with higher educational status more frequently experience hypersomnia and loss of interest. This differential effect means that using symptom counts to measure severity (as we have done) may obscure the relationship between MDD and educational status, just as may happen if the effect of sex is ignored (Coryell et al., 1992). Thus part of the difficulty in finding consistent relationships between MDD and educational status may reflect difficulties in identifying appropriate measures for analysis.

We made one further observation that tests models of how education influences MDD that has rarely been explored: the relationship between age of onset and education for patients with major depression. Consistent with the observation in the US that early episodes of MDD interfere with educational attainment, (Kessler et al., 1995) a later age of onset of MDD is associated with more years of education.

In interpreting our results it is important to realize that our samples were identified because they sought treatment. It is therefore possible that some of our results arise because of an ascertainment bias: a particular set of clinical features in MDD may be associated with help-seeking behavior. That is, it might be that the threshold for treatment seeking differs as a function of education. If more educated individuals seek treatment when the symptoms are not so severe, this could explain some of our results. But, as noted above, this cannot explain the full picture of our results (e.g. age at onset, hypersomnia and apathy being related positively to educational status) and so seems an implausible explanation for all of our findings. While we cannot exclude the possibility that such a bias exists we believe it is unlikely to substantially affect our results. Finally our results were obtained in a Han Chinese female population. We do not know if they similar features will be found in males, or to what extent the heterogeneity we describe here is typical of other, non-Chinese settings.

## Role of funding source

Funding for this study was provided by the Wellcome Trust; the Wellcome Trust had no further role in study design; in the collection, analysis and interpretation of data; in the writing of the report; and in the decision to submit the paper for publication.

## Conflict of interest

All authors declare they have no conflicts of interest including any financial, personal or other relationships with other people or organizations within three years of beginning the work submitted that could inappropriately influence, or be perceived to influence, their work.

## Figures and Tables

**Table 1:** Results of logistic regression of education on twenty-six symptoms of major depressive disorder.

Symptom	Wald	P-value	OR
Low appetite	9.36	0.002	0.87
Weight loss	0.38	0.537	0.98
Increased appetite	0.76	0.383	0.95
Weight gain	0.22	0.64	0.97
Insomnia	4.55	0.033	0.87
Hypersomnia	5.59	0.018	1.12
Slow speech or movement	6.93	0.008	0.90
Agitation	40.09	< 0.001*	0.78
Lack of energy	8.31	0.004	0.82
Worthless	8.21	0.004	0.88
Guilty	5.25	0.022	0.92
Distracted	1.22	0.27	1.06
Slowed thoughts	4.20	0.04	0.90
Hesitation	8.01	0.005	0.88
Thoughts of death	13.70	< 0.001*	0.86
Suicidal idea	20.06	< 0.001*	0.85
Suicidal plan	5.34	0.021	0.92
Lack of interest	27.03	< 0.001*	1.20
Unhappiness	6.61	0.01	0.88
Irritability	29.44	< 0.001*	0.80
Hopeless	5.19	0.023	0.90
Crying	0.02	0.896	1.00
Helpless	0.76	0.384	1.05
Nervous, jittery or anxious	2.29	0.13	1.10
Diurnal mood variation	1.33	0.248	0.96
Early waking	12.87	< 0.001*	0.83

The odds ratio (OR) shows the per year effect of education (so that an OR less than one indicates a reduced effect for those with higher educational status). In the P-value column an * indicates those results that are significant after applying a Bonferroni correction for multiple testing.

**Table 2:** Comorbid disorders in major depressive disorder and educational level.

Disorder	Wald	P value	OR
Melancholia	15.11	< 0.001*	0.83
GAD (generalized anxiety disorder)	0.12	0.73	0.99
Dysthymia	0.01	0.92	1.00
Panic disorder	1.00	0.31	0.94
Agoraphobia	0.20	0.66	1.02
Social phobia	0.29	0.59	0.98
Animal phobia	0.22	0.64	1.02
Situational phobia	2.43	0.12	0.94
Blood phobia	0.28	0.60	0.98

The odds ratio (OR) shows the per year effect of education (so that an OR less than one indicates a reduced effect for those with higher educational status). In the P-value column an * indicates those results that are significant after applying a Bonferroni correction for multiple testing.
